# Neuroprotective effects of sinomenine and metformin in diabetic stroke: Role of NLRP3/caspase‐1 and mitophagy

**DOI:** 10.1113/EP093135

**Published:** 2025-10-19

**Authors:** Wendi Li, Yiying Fan, Shaik Althaf Hussain

**Affiliations:** ^1^ Department of Neurology Harbin 242 Hospital Harbin China; ^2^ Health Service and Management Harbin Medical University Daqing China; ^3^ Department of Zoology, College of Science King Saud University Riyadh Saudi Arabia

**Keywords:** cerebral ischaemia–reperfusion injury, combination therapy, diabetes mellitus, metformin, neuroprotection, sinomenine

## Abstract

Type 2 diabetes mellitus (T2DM) significantly increases the risk and severity of cerebral ischaemia–reperfusion (IR) injury, yet effective neuroprotective treatments for diabetic stroke remain limited. This study explored whether a combination of sinomenine and metformin could offer enhanced neuroprotection in diabetic rats subjected to cerebral IR injury, and investigated the molecular pathways involved. Male rats were rendered diabetic using a high‐fat diet and low‐dose streptozotocin. After establishing diabetes, rats underwent transient middle cerebral artery occlusion followed by 24 h of reperfusion. Animals received sinomenine, metformin, or their combination for 7 days prior to IR induction. Neurological function was assessed using standardized behavioural tests. Molecular analyses measured markers of pyroptosis (NLRP3, cleaved caspase‐1, gasdermin D N‐terminal fragment), mitophagy (PTEN‐induced kinase 1 (PINK‐1), Parkin), inflammation (interleukin (IL)‐1β, IL‐18) and oxidative stress (malondialdehyde, superoxide dismutase, glutathione peroxidase). To determine the role of mitophagy, a subset of animals was pretreated with the mitophagy inhibitor mitochondrial division inhibitor 1 (Mdv‐1). Combination therapy led to significant improvements in neurological performance, accompanied by downregulation of pyroptosis‐associated proteins and pro‐inflammatory cytokines, as well as enhanced activation of the PINK‐1/Parkin mitophagy pathway and improved antioxidant status. The neuroprotective effects of the combined treatment were abolished by Mdv‐1, indicating a critical role for mitophagy in mediating these benefits. The combination of sinomenine and metformin confers additive neuroprotection against cerebral IR injury in diabetic rats, primarily through inhibition of NLRP3‐mediated pyroptosis and activation of the PINK‐1/Parkin mitophagy pathway. These findings highlight a promising therapeutic strategy for diabetic stroke and warrant further preclinical and clinical investigation.

## Abstract (highlights)

- **What is the central question of this study?**
  Can a combination of sinomenine and metformin enhance neuroprotection in diabetic rats subjected to cerebral ischaemia–reperfusion injury, and what molecular pathways mediate these effects?
- **What is the main finding and its importance?**
  Combined therapy significantly reduced pyroptosis and inflammation while enhancing mitophagy and antioxidant defences. The protective effects were dependent on PINK‐1/Parkin‐mediated mitophagy. These findings suggest a promising additive approach for improving stroke outcomes in patients with type 2 diabetes.

## 1 INTRODUCTION

Cerebral ischaemia–reperfusion (IR) injury is a critical pathological event that occurs when blood supply to the brain is interrupted and subsequently restored, leading to a cascade of cellular and molecular disturbances. This process results in oxidative stress, calcium overload, inflammation and ultimately neuronal death, contributing significantly to morbidity and mortality worldwide. Among various risk factors, type 2 diabetes mellitus (T2DM) plays a pivotal role in exacerbating cerebral IR injury (Kilic & Halici, 2025). Diabetes induces chronic metabolic disturbances such as hyperglycaemia, insulin resistance and dyslipidaemia, which amplify oxidative stress and inflammatory responses in the brain. These alterations not only increase the susceptibility of neural tissue to ischaemic damage but also impair endogenous repair mechanisms, thereby intensifying neuronal injury and worsening functional outcomes after stroke. Moreover, diabetes diminishes the brain's responsiveness to neuroprotective therapies, posing a significant challenge in clinical management (P.‐F. Ding et al., 2022). Therefore, the development of effective therapeutic strategies specifically targeting cerebral IR injury in diabetic patients is of paramount importance, as it holds the potential to improve neurological outcomes, reduce disability and ultimately decrease mortality in this high‐risk population (Soares et al., 2019).

Pyroptosis is an inflammatory form of programmed cell death that is distinct from apoptosis and necrosis, and it plays a crucial role in the pathogenesis of cerebral IR injury, especially under diabetic conditions (Zheng et al., 2022). This process is primarily mediated by the NOD‐like receptor family pyrin domain‐containing 3 (NLRP3) inflammasome, a multiprotein complex in which NLRP3 acts as a sensor for cellular stress, and caspase‐1 functions as an effector enzyme that activates pro‐inflammatory cytokines and induces cell death (Ghafouri‐Fard et al., 2022). In diabetes, chronic metabolic disturbances such as hyperglycaemia and increased oxidative stress enhance the activation of the NLRP3–caspase‐1 inflammasome axis, leading to excessive pyroptosis and the release of inflammatory mediators. This not only exacerbates neuronal injury following cerebral IR but also amplifies neuroinflammation and impairs recovery (X. Li et al., 2022; Yu et al., 2020). Mitophagy, a selective form of autophagy that removes damaged mitochondria, plays a critical role in regulating pyroptosis by maintaining mitochondrial quality and limiting NLRP3 inflammasome activation. Impaired mitophagy in diabetic IR injury can therefore exacerbate neuronal damage and inflammation (Gong et al., 2022; Matsuda & Tanaka, 2010). Together, dysregulated mitophagy and excessive pyroptosis form a critical pathological axis in diabetic cerebral IR injury, representing a promising target for neuroprotective interventions.

Despite numerous therapeutic interventions demonstrating neuroprotection in animal models of cerebral IR injury, successful translation to clinical practice remains elusive. One major limitation is that preclinical studies frequently employ healthy animals, overlooking the presence of comorbidities such as obesity, hypertension and diabetes, which are highly prevalent among stroke patients. These comorbid conditions profoundly alter the pathophysiology of cerebral IR injury by disrupting endogenous protective signalling pathways, thereby increasing the brain's vulnerability to ischaemic damage and diminishing the efficacy of neuroprotective treatments (Xu & Pan, 2013). Moreover, cerebral IR injury is a complex and multifactorial process involving numerous overlapping molecular and cellular pathways. As a result, monotherapies that target a single mechanism are often insufficient to achieve meaningful neuroprotection. Recently, combination therapies that simultaneously modulate multiple pathological pathways have garnered considerable interest as potentially more effective strategies (Lapchak & Araujo, 2007). Sinomenine, a bioactive alkaloid with potent anti‐inflammatory and neuroprotective properties, has demonstrated efficacy in reducing infarct size, cerebral oedema and blood–brain barrier disruption in experimental models of cerebral IR injury (Pazoki‐Toroudi et al., 2016; Yang et al., 2016; Yao et al., 2024; Zhang et al., 2021). Metformin, a first‐line agent for T2DM, also exerts neuroprotective and anti‐inflammatory effects independent of its glucose‐lowering action, including benefits in ischaemic stroke models (Jia et al., 2015; J. Li et al., 2010). Given their distinct yet complementary mechanisms of action, the combination of sinomenine and metformin holds the potential to provide additive neuroprotection against cerebral IR injury, particularly in the setting of diabetes, where inflammation and metabolic dysfunction are pronounced. Notably, no previous studies have investigated this therapeutic combination in diabetic models of cerebral IR injury, highlighting a novel and promising direction for future research.

To the best of our knowledge, this is the first study to investigate the combined effects of sinomenine and metformin on cerebral IR injury in a diabetic model, with a particular emphasis on NLRP3‐mediated pyroptosis and mitophagy. By incorporating diabetes as a major comorbidity, this study aims to address an underexplored area in preclinical stroke research. We hypothesized that combined sinomenine and metformin therapy would attenuate cerebral IR injury in diabetic rats by suppressing NLRP3‐mediated pyroptosis and enhancing mitophagy.

## 2 METHODS

### 2.1 Ethical approval

All procedures involving animals were carried out in compliance with the ethical standards and policies of *Experimental Physiology* concerning animal research. Approval for the experimental protocols was obtained from the Harbin 242 Hospital Animal Experiment Ethics Review Committee, China (Ethics Code: A2024000933). The investigations were conducted in full accordance with the *Guide for the Care and Use of Laboratory Animals* (8th Edition, 2011, NIH) and were reported following the ARRIVE 2.0 guidelines. Throughout the study, animal welfare was carefully safeguarded, and every effort was made to reduce pain and distress. Euthanasia was conducted in line with the AVMA Guidelines for the Euthanasia of Animals.

### 2.2 Experimental animals

Sixty‐six male Sprague–Dawley rats (200–250 g, 8–10 weeks old) were obtained from the institutional animal facility and housed under standard conditions (20–24°C, 55% humidity, 12 h light/dark cycle) with ad libitum access to chow and water. An a priori power analysis was performed, assuming a large effect size (*f* = 0.40), α = 0.05 and power = 0.80, which indicated a minimum requirement of approximately six rats per group, resulting in a total of 66 animals. Six rats were maintained on a regular diet for 12 weeks to serve as non‐diabetic controls, while the remaining 60 rats underwent induction of T2DM over the same period. Following confirmation of diabetes, the diabetic rats were randomly assigned to five experimental groups within two protocols (neurobehavioural and mechanistic) using a computer‐generated random number sequence to ensure unbiased allocation. All surgical procedures, drug administrations and outcome assessments (behavioural and molecular) were performed by investigators blinded to the treatment groups to minimize bias.

### 2.3 Development of the diabetic rat model

To generate a rat model that closely mimics human T2DM, a two‐step protocol was employed. Following a 2‐week acclimatization period, rats were transitioned to a high‐fat diet formulated to provide approximately 60% of total caloric intake from fat sources, including lard and cholesterol. Specifically, the diet consisted of the base chow supplemented with 8% lard, 1% pure cholesterol and 0.05% sodium cholate by weight. This dietary regimen was maintained for 6 weeks to induce insulin resistance and metabolic alterations characteristic of T2DM. At the start of the seventh week, animals underwent an overnight fast and subsequently received a single intraperitoneal injection of streptozotocin (STZ) at a dose of 35 mg/kg, freshly prepared in citrate buffer (pH 4.5). This low‐dose STZ administration was intended to partially impair pancreatic β‐cell function, further promoting hyperglycaemia in the context of pre‐existing insulin resistance. Seventy‐two hours after STZ injection, blood glucose concentrations were measured from tail vein samples using a calibrated glucometer. Rats exhibiting fasting blood glucose levels above 250 mg/dL were considered diabetic and included in the study, while those below this threshold were excluded. The diabetic state was maintained for an additional 6 weeks, during which body weights were monitored weekly. Throughout the experiment, non‐diabetic control rats were maintained on a standard chow diet. This approach ensured the establishment of a stable T2DM phenotype prior to allocation into experimental groups for subsequent interventions and cerebral IR procedures.

### 2.4 Experimental group allocation and treatment paradigms

(1) *Neurobehavioural Assessment Protocol*: To evaluate the impact of different therapeutic regimens on neurological function after cerebral IR, diabetic rats were randomly distributed into five groups (*n* = 6 per group, total *n* = 30). The experimental arms were as follows:

(i) Sham: Animals underwent all surgical steps except for the induction of middle cerebral artery occlusion (MCAO) and received no drug treatment.
(ii) IR: Rats were subjected to 30 min of transient MCAO followed by 24 h of reperfusion without pharmacological intervention.
(iii) IR+Sin: Sinomenine was dissolved in a mixture of 10% dimethyl sulfoxide, 40% polyethylene glycol 300, 5% Tween 80 and 45% saline, and administered intraperitoneally at 80 mg/kg/day for seven consecutive days prior to IR induction (Xia et al., 2022).
(iv) IR+Met: Metformin was dissolved in sterile saline and given intraperitoneally at 30 mg/kg/day for 7 days before surgery (Ruan et al., 2021).
(v) IR+Sin+Met: Both agents were administered concurrently at the above doses and schedule.

The doses of sinomenine and metformin used in this study were selected based on previously published studies demonstrating optimal efficacy in rat models of cerebral IR injury (Ruan et al., 2021; Xia et al., 2022). Following the completion of a battery of established behavioural tests 24 h after reperfusion or sham surgery – including neurological deficit scoring, the corner test, and the adhesive removal test – the rats were humanely euthanized in accordance with the AVMA Guidelines for the Euthanasia of Animals. Euthanasia was performed under deep anaesthesia induced by an intraperitoneal injection of ketamine (60 mg/kg) and xylazine (10 mg/kg), followed by decapitation to ensure rapid and humane death.

(2) *Mechanistic Investigation Protocol*: A separate cohort of diabetic rats (*n* = 30) was dedicated to exploring the cellular and molecular mechanisms underlying the observed neuroprotective effects, with a particular focus on pyroptosis and mitophagy pathways. Based on initial findings favouring combination therapy, this phase omitted monotherapy groups and instead concentrated on the effects of mitophagy inhibition. The animals were divided as follows (*n* = 6 per group):

(i) Sham: Same as in the first protocol.
(ii) IR: Same as in the first protocol.
(iii) IR+Sin+Met: Sinomenine and metformin were co‐administered as described in the first protocol.
(iv) IR+Mdv‐1: Mitochondrial division inhibitor 1 (Mdv‐1) was administered intraperitoneally at 1 mg/kg/day for 7 days before surgery to selectively inhibit mitophagy (C. Ding & Zhang, 2023).
(v) IR+Sin+Met+Mdv‐1: Rats received both sinomenine and metformin as well as Mdv‐1, following the above protocols.

In the Mechanistic Investigation Protocol, 24 h after reperfusion or sham surgery, rats were deeply anaesthetized with an intraperitoneal injection of ketamine (60 mg/kg) and xylazine (10 mg/kg). Euthanasia was subsequently performed by decapitation under deep anaesthesia, in accordance with the AVMA Guidelines for the Euthanasia of Animals to ensure minimal suffering. The brains were then rapidly removed, and the hippocampus was carefully dissected using specialized instruments for subsequent molecular and biochemical analyses.

### 2.5 Evaluation of metabolic parameters in diabetic rats

Fasting blood sugar (FBS) concentrations were measured using a digital glucometer (Convergent Technologies, Cölbe, Germany) after an overnight fast. Plasma insulin levels were quantified via a commercially available enzyme‐linked immunosorbent assay (ELISA) kit (Cayman Chemical Co., Ann Arbor, MI, USA), following the manufacturer's protocol. Insulin resistance was estimated by calculating the homeostasis model assessment of insulin resistance (HOMA‐IR) index, using the formula: (fasting insulin [µU/mL] × fasting glucose [mmol/L])/22.5. Additionally, total cholesterol levels in plasma were determined with an ELISA kit obtained from Thermo Fisher Scientific (Waltham, MA, USA), according to the supplier's instructions. These assessments were performed to confirm the establishment of metabolic disturbances characteristic of T2DM in the experimental animals.

### 2.6 Surgical induction of focal cerebral ischaemia

To model transient focal cerebral ischaemia, rats assigned to the IR groups underwent MCAO using the intraluminal filament technique. Anaesthesia was induced via intraperitoneal injection of ketamine (60 mg/kg) and xylazine (10 mg/kg). After confirming adequate anaesthesia, the ventral neck region was shaved and disinfected, and a midline incision was made to expose the carotid artery complex under a surgical microscope. The right common, external, and internal carotid arteries were carefully separated. Temporary ligatures were placed on the common and internal carotid arteries to control blood flow. The external carotid artery was ligated, and a small arteriotomy was created. A nylon monofilament with a rounded tip was gently introduced through the external carotid artery stump and advanced along the internal carotid artery until resistance indicated occlusion of the origin of the middle cerebral artery. The filament remained in position for 30 min to induce ischaemia, after which it was withdrawn to allow reperfusion for 24 h. Sham‐operated animals underwent the same surgical exposure and vessel manipulation without filament insertion. Following surgery, incisions were sutured and animals were monitored in a temperature‐controlled environment during recovery. Throughout the procedure, meticulous care was taken to maintain body temperature and minimize surgical stress. Postoperative analgesia was provided with buprenorphine (0.05 mg/kg, subcutaneously) immediately after surgery to ensure animal welfare. The success of MCAO was confirmed by the presence of neurological deficits postoperatively. Animals were excluded from analysis if MCAO failed or if perioperative mortality occurred; in the present study, eight rats were excluded due to mortality following MCAO.

### 2.7 Evaluation of neurological outcomes

All behavioural outcome assessments were conducted by investigators blinded to group allocations in order to minimize observer bias.

(1) *Neurological Deficit Scoring*: To quantify neurological impairment 24 h after reperfusion, a modified Longa scoring system was employed, ranging from 0 to 4 points:

Score 0: No observable neurological deficit; normal behaviour.

Score 1: Incomplete extension of the left forelimb.

Score 2: Spontaneous circling to the left (contralateral to the lesion) when walking.

Score 3: Tendency to fall or tip toward the left side during ambulation.

Score 4: No spontaneous motor activity and/or decreased level of consciousness. Each rat was observed and scored by an investigator blinded to group assignments.

(2) *Corner Test*: The corner test was utilized to detect asymmetries in sensorimotor function and postural control. Rats were gently guided into a corner formed by two boards positioned at a 30° angle. Upon entering the corner, whisker stimulation encouraged the animal to rear and turn. Healthy rats typically turned equally to both sides, whereas animals with unilateral brain injury showed a preference for turning toward the ipsilateral (injured) side. Each animal underwent 10 consecutive trials, and the percentage of turns toward the affected side was calculated to assess lateralized deficits.
(3) *Adhesive Removal Test*: To assess somatosensory function and fine motor coordination, the adhesive removal test was performed 24 h post‐reperfusion. Small adhesive dots were placed on the palmar surface of each forepaw. Rats were then placed individually in a transparent enclosure, and the time required to detect and remove each adhesive was recorded, with a maximum cutoff of 120 s per trial to minimize distress. Prolonged removal times indicated impaired sensory or motor function, while shorter times reflected functional recovery. Each forelimb was tested separately, and the results were averaged for analysis.

### 2.8 Protein expression profiling

Following dissection, hippocampal tissue samples were promptly frozen at −80°C for preservation until analysis. For protein extraction, samples were homogenized in lysis buffer using a commercial protein extraction kit (KeyGEN BioTECH, cat. no. KGP902, Nanjing, China), and the resulting lysates were quantified for total protein concentration. Equal amounts of protein from each sample were loaded onto 8% SDS–polyacrylamide gels for electrophoretic separation. Proteins were then transferred onto polyvinylidene difluoride membranes using a semi‐dry transfer apparatus. Membranes were blocked for 20 min in a rapid blocking solution to prevent non‐specific binding, followed by overnight incubation at 4°C with specific primary antibodies targeting NLRP3 (1:1000, Abcam, Waltham, MA, USA), cleaved caspase‐1 (1:2000, Thermo Fisher Scientific, Waltham, MA, USA), pro‐caspase‐1 (1:2000, Thermo Fisher Scientific, Waltham, MA, USA), gasdermin D N‐terminal fragment (GSDMD‐N; 1:1000, Cell Signaling Technology, Danvers, MA, USA), PTEN‐induced kinase 1 (PINK‐1; 1:1000, Abcam, Waltham, MA, USA), Parkin (1:1000, Abcam, Waltham, MA, USA) and β‐actin (1:1000, Abcam, Waltham, MA, USA) as a loading control. After primary antibody incubation, membranes were washed and then exposed to horseradish peroxidase‐conjugated goat anti‐rabbit IgG secondary antibody (1:1000, Cell Signaling Technology, Danvers, MA, USA) for 1 h at room temperature. Detection of immunoreactive bands was accomplished using an enhanced chemiluminescence (ECL) system (Bio‐Rad Laboratories, Hercules, CA, USA). Densitometric analysis of protein bands was performed with ImageJ software, and all target protein levels were normalized to β‐actin to ensure accurate quantification.

### 2.9 Quantification of inflammatory and oxidative stress biomarkers

For the assessment of inflammatory mediators and oxidative stress parameters, hippocampal tissue samples were first homogenized in ice‐cold phosphate‐buffered saline. The homogenates were then centrifuged at 1400 *g* for 10 min at 4°C to separate the cellular debris. The resulting supernatants were collected and stored on ice for immediate analysis. Levels of the pro‐inflammatory cytokines interleukin (IL)‐1β and IL‐18 were quantified using commercially available ELISA kits (NeoBioscience, Shenzhen, China), following the manufacturer's instructions. Calibration curves were generated using known standards supplied with each kit, allowing for precise determination of cytokine concentrations in the tissue samples. To evaluate oxidative stress status, the concentrations of malondialdehyde (MDA), superoxide dismutase (SOD) and glutathione peroxidase (GPx) were measured using specific colorimetric assay kits (Zcibio Co., Shanghai, China). MDA levels served as an index of lipid peroxidation, while SOD and GPx activities reflected the tissue's antioxidant defence capacity. All measurements were standardized to total protein content, which was determined using a bicinchoninic acid (BCA) protein assay. Final results for MDA were expressed as micromoles per milligram of protein, while SOD and GPx activities were expressed as units per milligram of protein.

### 2.10 Data processing and statistical evaluation

All quantitative data were analysed using GraphPad Prism (version 9.5.1, GraphPad Software, Boston, MA, USA) on a Windows platform. Continuous variables are presented as mean ± standard deviation (SD). Comparisons between two independent groups were performed using an unpaired Student's *t*‐test, while differences among more than two groups were evaluated by one‐way ANOVA followed by Tukey's multiple comparison test. A *P*‐value <0.05 was considered statistically significant. Prior to hypothesis testing, data distributions were visually inspected, and normality was formally assessed using the Shapiro–Wilk test to ensure the appropriateness of parametric analyses.

## 3 RESULTS

### 3.1 Assessment of key metabolic indicators in diabetic rats

**FIGURE 1:**
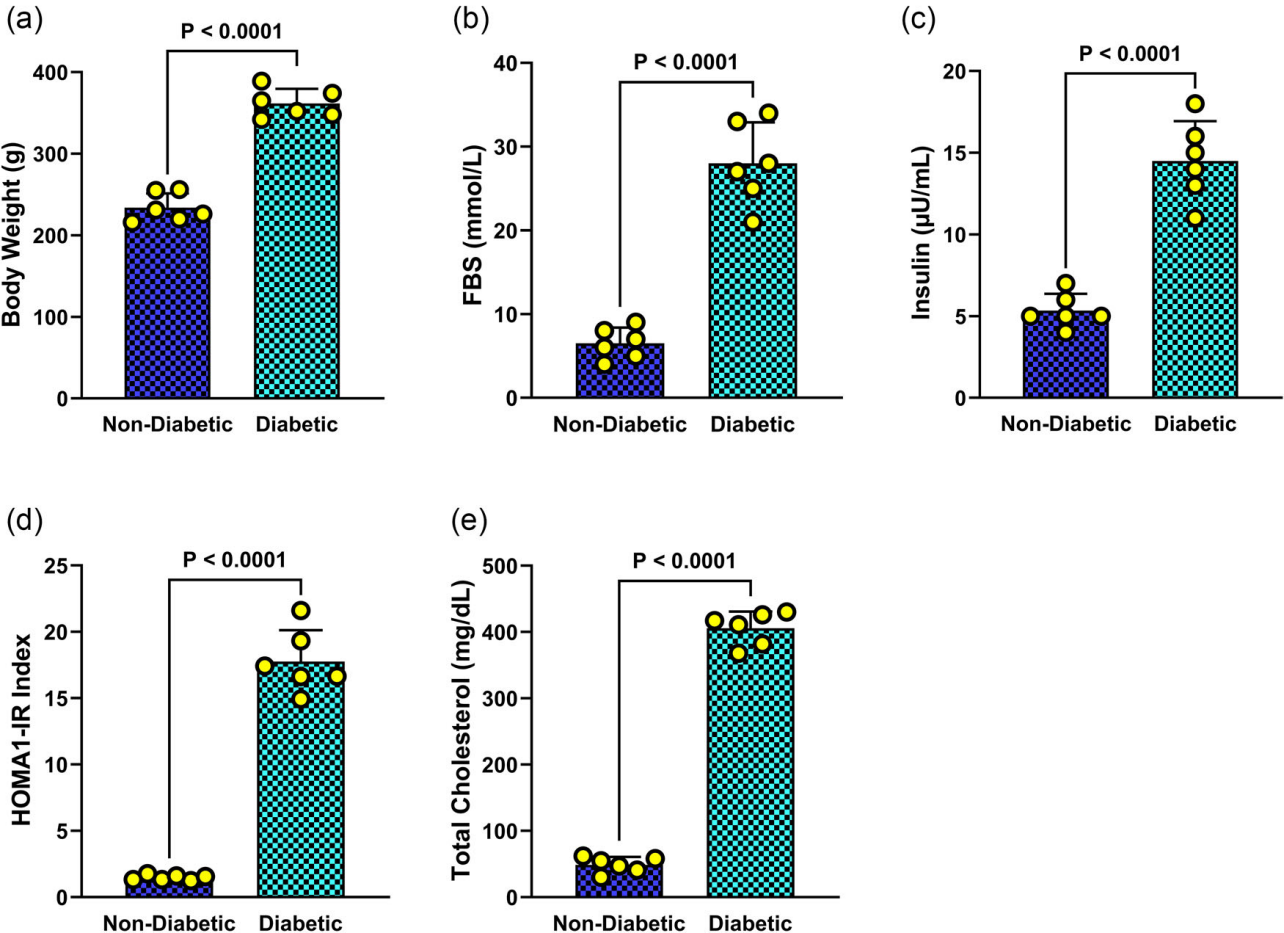
Comparative assessment of metabolic parameters in control and diabetic rats. (a) Final body weight, (b) fasting blood glucose (FBS), (c) plasma insulin concentration, (d) homeostatic model assessment of insulin resistance (HOMA‐IR) index, and (e) total plasma cholesterol levels measured at the end of the experimental period. Data are presented as means ± SD (*n* = 6 per group). Statistical significance between groups was determined using Student's *t*‐test, with *P* < 0.05 considered significant.

Upon completion of the 12‐week induction period, comprehensive metabolic profiling revealed marked differences between diabetic and non‐diabetic rats. Statistical analysis demonstrated that diabetic animals exhibited significantly greater body weight, FBS, plasma insulin concentrations, HOMA‐IR values, and total cholesterol levels compared to controls (all *P* < 0.0001; Figure 1a–e). These pronounced alterations in metabolic parameters confirm the successful establishment of the T2DM model and underscore the extent of metabolic dysregulation present in the diabetic cohort.

### 3.2 Additive neuroprotection by combined sinomenine and metformin in diabetic IR injury

**FIGURE 2:**
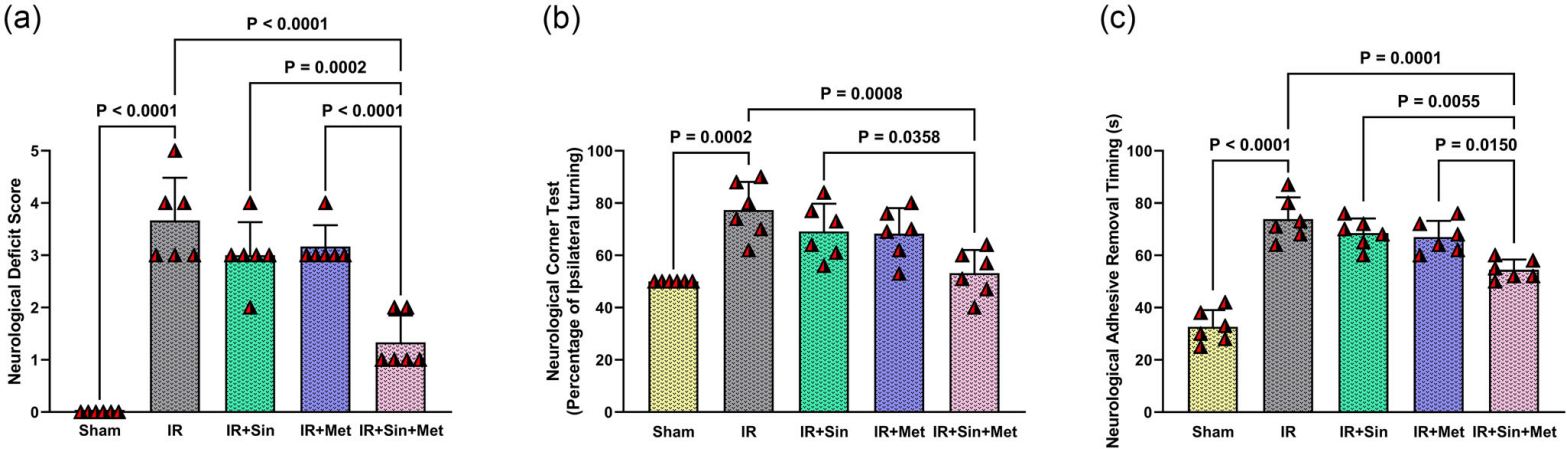
Evaluation of neurological outcomes following cerebral IR and different treatment regimens in diabetic rats. (a) Neurological deficit scores were assessed using a standardized scoring system. (b) Sensorimotor function was evaluated by the corner test. (c) Somatosensory response measured as adhesive removal latency. Data are expressed as means ± SD (*n* = 6 per group). Statistical comparisons among groups were performed using one‐way ANOVA followed by Tukey's *post hoc* test. IR, ischaemia–reperfusion; Met, metformin; Sin, sinomenine.

Figure 2a demonstrates that rats in the Sham group exhibited normal neurological function, consistently receiving a score of zero, indicative of the absence of any deficits. In contrast, animals subjected to IR displayed pronounced neurological impairments, such as impaired forelimb extension and a tendency to veer or tip toward the affected side during movement, resulting in significantly elevated scores compared to the Sham group (*P* < 0.0001). Administration of either sinomenine or metformin alone prior to IR did not lead to a statistically meaningful improvement in these neurological scores. Remarkably, rats pretreated with the combination of sinomenine and metformin showed a substantial reduction in neurological deficit scores relative to the IR group (*P* < 0.0001) and when compared to either monotherapy (sinomenine: *P* = 0.0002; metformin: *P* < 0.0001), highlighting an additive effect of the combined regimen (Figure 2a).

A similar pattern emerged in the corner test (Figure 2b), where IR animals exhibited a marked increase in lateralized turning behaviour compared to Sham controls (*P* = 0.0002). Neither sinomenine nor metformin alone significantly ameliorated this behavioural abnormality. However, the dual therapy group demonstrated a significant decrease in abnormal turning, both versus the IR group (*P* = 0.0008) and in comparison with sinomenine monotherapy (*P* = 0.0358) (Figure 2b).

Consistent findings were observed in the adhesive removal test (Figure 2c). IR rats required significantly longer times to detect and remove adhesive patches from their forepaws compared to Sham animals (*P* < 0.0001). Single‐agent treatments did not significantly shorten adhesive removal times. In contrast, combined pretreatment with sinomenine and metformin led to a pronounced reduction in removal latency relative to the IR group (*P* = 0.0001) and to both sinomenine (*P* = 0.0055) and metformin (*P* < 0.0150) monotherapies (Figure 2c).

Collectively, these results underscore the enhanced neuroprotective efficacy of the sinomenine and metformin combination, which outperforms either agent alone in mitigating neurological deficits following cerebral IR injury in diabetic rats.

### 3.3 Dual therapy reduces IR‐induced pyroptosis in diabetic brains, but this benefit is reversed by blocking mitophagy

**FIGURE 3:**
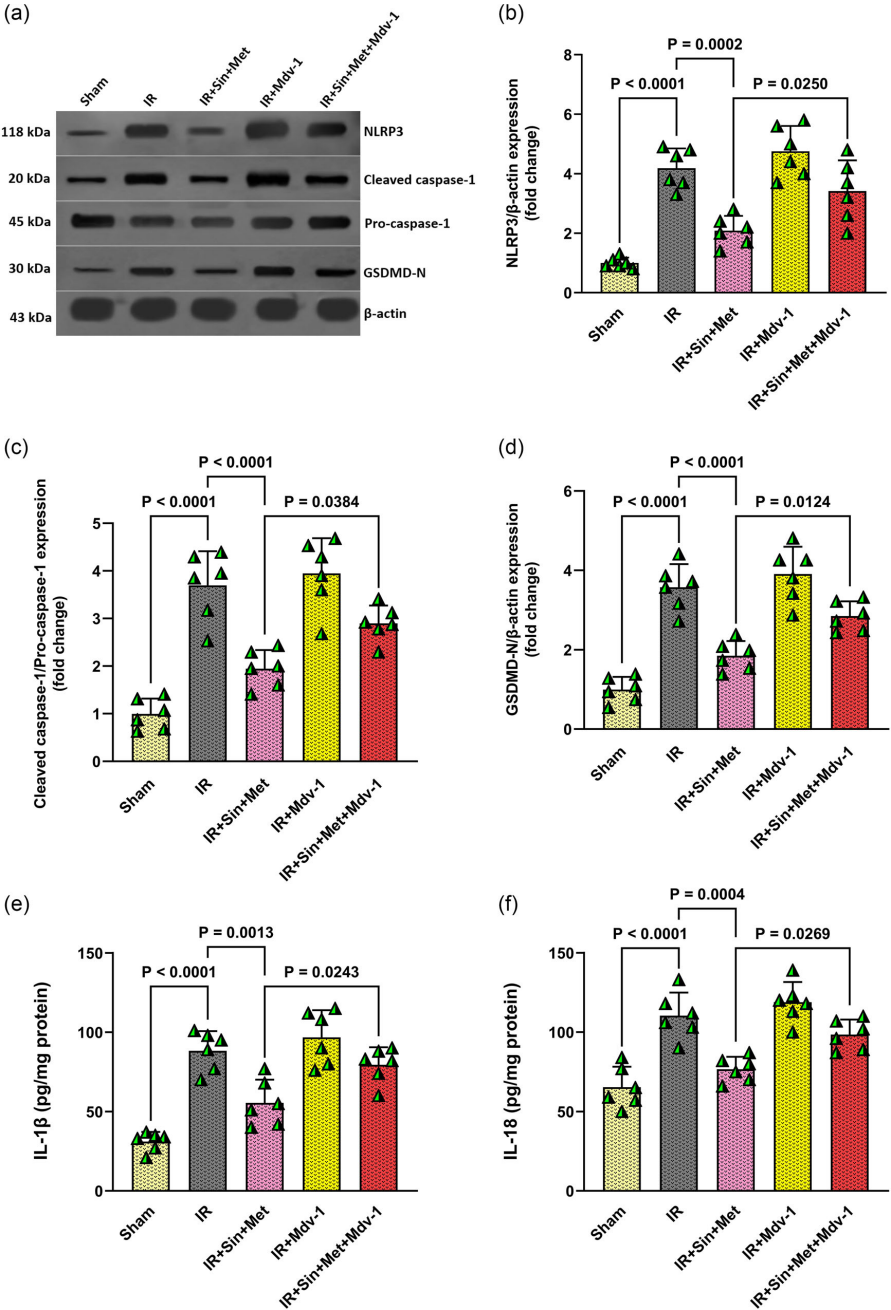
Effect of combination therapy and mitophagy inhibition on pyroptosis and inflammatory mediators in diabetic rats following cerebral IR. (a) Representative western blot images showing protein levels of NLRP3, cleaved caspase‐1, and GSDMD‐N. (b–d) Densitometric quantification of NLRP3, cleaved caspase‐1, and GSDMD‐N normalized to β‐actin. (e, f) ELISA measurement of pro‐inflammatory cytokines IL‐1β and IL‐18 in brain tissue. Data are presented as means ± SD (*n* = 6 per group). Statistical comparisons among groups were performed using one‐way ANOVA followed by Tukey's *post hoc* test. IR, ischaemia–reperfusion; Mdv‐1, mitochondrial division inhibitor‐1; Met, metformin; Sin, sinomenine.

Building on the observed superior neuroprotection of combined sinomenine and metformin therapy, we next explored the molecular mechanisms underlying this effect, with a particular focus on pyroptosis and the role of mitophagy. To this end, the mitophagy inhibitor Mdv‐1 was administered in conjunction with the combination therapy to assess its impact on neuroprotection. Western blot analyses demonstrated that IR in diabetic rats resulted in marked upregulation of pyroptosis‐associated proteins, including NLRP3, cleaved caspase‐1 and GSDMD‐N, compared to Sham‐operated controls (all *P* < 0.0001; Figure 3a–d). Treatment with the combination of sinomenine and metformin significantly attenuated the expression of these proteins (NLRP3: *P* = 0.0002; cleaved caspase‐1 and GSDMD‐N: both *P* < 0.0001 vs. IR group), indicating a robust suppression of pyroptotic cell death. However, when Mdv‐1 was co‐administered with the combination therapy, the levels of NLRP3, cleaved caspase‐1 and GSDMD‐N were significantly elevated compared to the combination therapy group alone (*P* = 0.0250, *P* = 0.0384 and *P* = 0.0124, respectively), suggesting that inhibition of mitophagy partially reverses the anti‐pyroptotic benefits of the dual treatment (Figure 3a–d).

Consistent with these findings, ELISA results revealed that IR injury led to a significant rise in the pro‐inflammatory cytokines IL‐1β and IL‐18 relative to the Sham group (both *P* < 0.0001; Figure 3e, f). Combination therapy markedly reduced the levels of both cytokines (IL‐1β: *p* = 0.0013; IL‐18: *P* = 0.0004 vs. IR group). Notably, the addition of Mdv‐1 diminished the anti‐inflammatory effect, as evidenced by higher IL‐1β and IL‐18 concentrations compared to combination therapy alone (*P* = 0.0243 and *P* = 0.0269, respectively).

Overall, these results indicate that the neuroprotective and anti‐pyroptotic actions of combined sinomenine and metformin therapy in diabetic IR injury are, at least in part, dependent on intact mitophagy pathways. Inhibition of mitophagy undermines these beneficial effects, highlighting the importance of mitochondrial quality control in modulating pyroptosis and inflammation in the diabetic brain following ischaemic insult.

### 3.4 Dual therapy promotes mitophagy in diabetic ischaemic brains, an effect attenuated by mitophagy blockade

**FIGURE 4:**
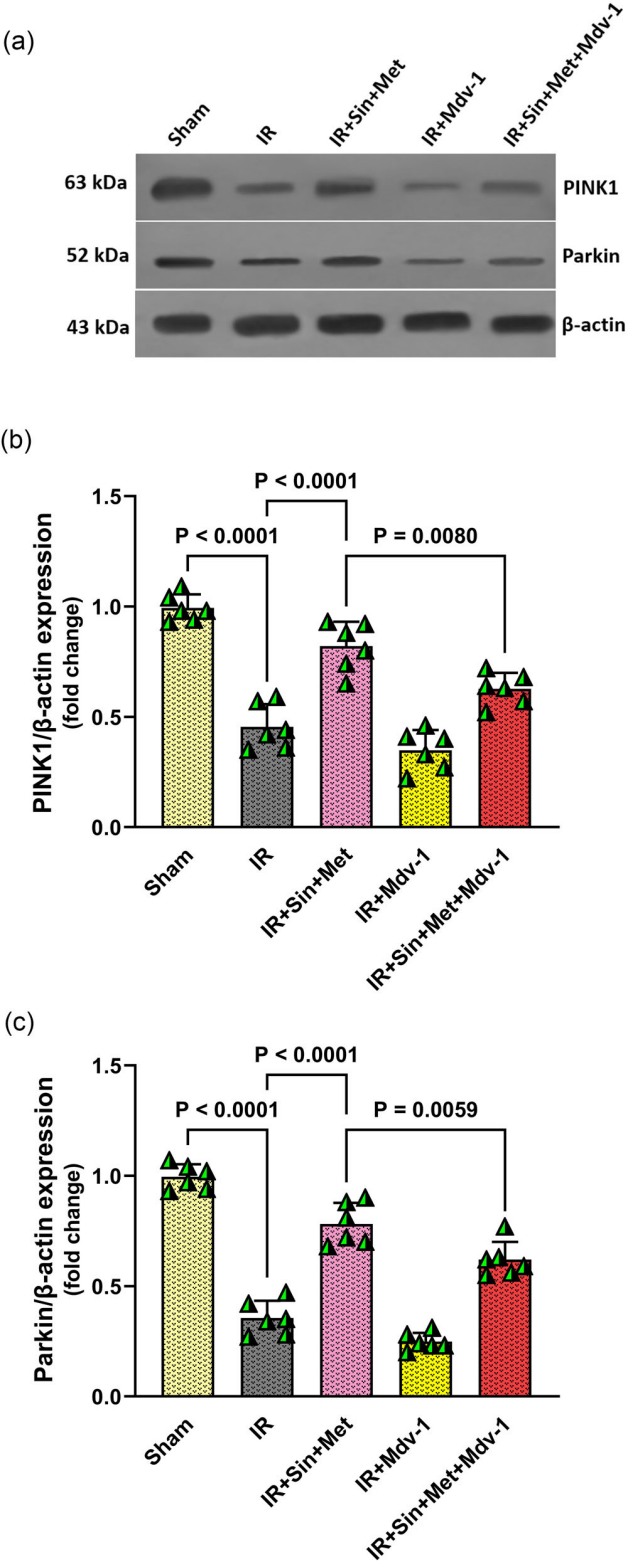
Effect of combination therapy and mitophagy inhibition on mitophagy‐related proteins in diabetic rats following cerebral IR. (a) Representative western blot images showing PINK1 and Parkin protein expression in brain tissue. (b, c) Densitometric quantification of PINK1 and Parkin normalized to β‐actin. Data are expressed as means ± SD (n = 6 per group). Statistical analysis was performed using one‐way ANOVA followed by Tukey's *post hoc* test. IR, ischaemia–reperfusion; Mdv‐1, mitochondrial division inhibitor‐1; Met, metformin; Sin, sinomenine.

To further elucidate the role of mitophagy in the observed neuroprotection, we assessed the expression of key mitophagy regulators, PINK‐1 and Parkin, in brain tissue from diabetic rats using western blot analysis (Figure 4a–c). Cerebral IR injury led to a marked reduction in both PINK‐1 and Parkin protein levels when compared to the Sham group (*P* < 0.0001 for both), indicating impaired mitophagy following IR insult. Administration of the combined sinomenine and metformin therapy significantly restored the expression of PINK‐1 and Parkin relative to the IR group (*P* < 0.0001 for both), suggesting that this dual treatment enhances mitophagy activity in the diabetic brain after IR injury. However, when Mdv‐1, a mitophagy inhibitor, was co‐administered with the combination therapy, the upregulation of PINK‐1 and Parkin was significantly blunted compared to the combination therapy group alone (*P* = 0.0080 and *P* = 0.0059, respectively) (Figure 4b, c). This finding confirms that the beneficial effect of combination therapy on mitophagy is dependent on intact mitophagic signalling.

### 3.5 Dual therapy alleviates IR‐induced oxidative stress in diabetic ischaemic brains, an effect attenuated by mitophagy blockade

**FIGURE 5:**
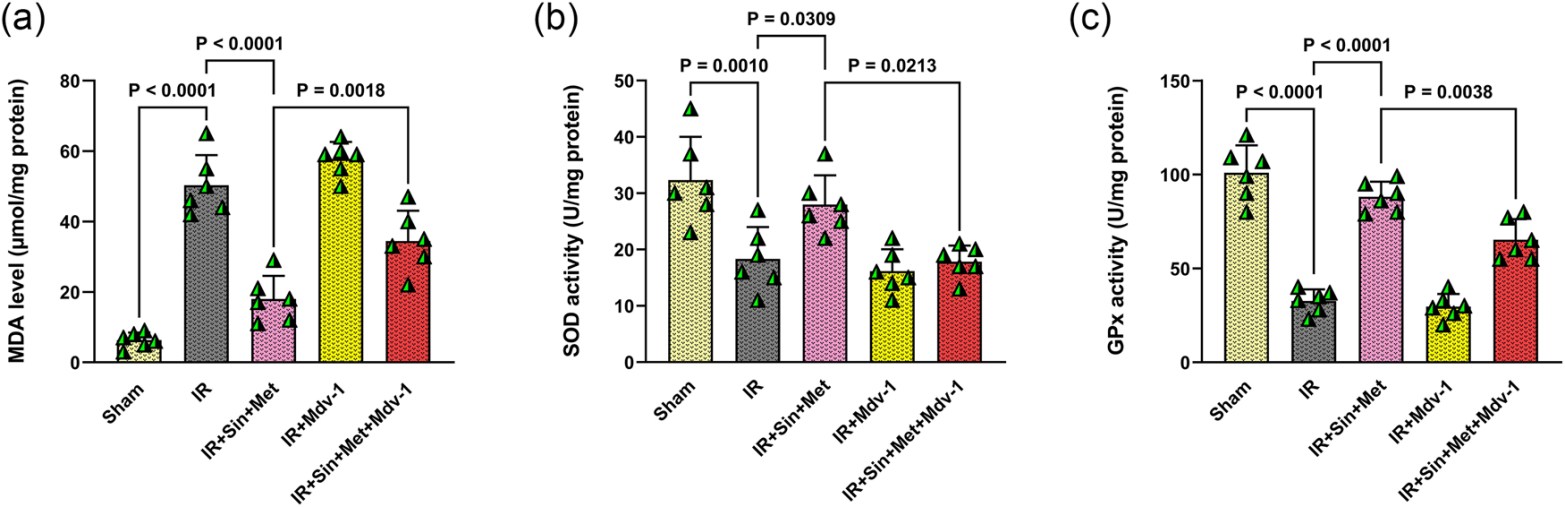
Effect of combination therapy and mitophagy inhibition on oxidative stress in diabetic rats following cerebral IR. (a) Malondialdehyde (MDA) levels, (b) superoxide dismutase (SOD) activity, and (c) glutathione peroxidase (GPx) activity were measured in brain tissue homogenates. Data are presented as mean ± SD (*n* = 6 per group). Statistical comparisons among groups were performed using one‐way ANOVA followed by Tukey's *post hoc* test. IR, ischaemia–reperfusion; Mdv‐1, mitochondrial division inhibitor‐1; Met, metformin; Sin, sinomenine.

Assessment of oxidative stress markers revealed that IR injury in diabetic rats led to a pronounced elevation in MDA levels (*P* < 0.0001), alongside significant reductions in the activities of the antioxidant enzymes SOD (*P* = 0.0010) and GPx (*P* < 0.0001), when compared to the Sham group (Figure 5a–c). Treatment with the combination of sinomenine and metformin markedly lowered MDA concentrations (*P* < 0.0001) and significantly enhanced both SOD (*P* = 0.0309) and GPx (*P* < 0.0001) activities relative to the IR group, indicating a strong antioxidative effect. The co‐administration of the mitophagy inhibitor Mdv‐1 with combination therapy abolished the antioxidant benefits, as evidenced by significantly increased MDA levels (*P* = 0.0018) and reduced SOD (*P* = 0.0213) and GPx (*P* = 0.0038) activities compared to combination therapy alone (Figure 5a–c). These findings underscore that the antioxidative benefits of dual therapy in diabetic IR brains are closely linked to functional mitophagy, and disruption of this pathway compromises the therapy's protective effects against oxidative stress.

## 4 DISCUSSION

In the present investigation, we explored the potential of combined sinomenine and metformin therapy to mitigate neurological damage in a diabetic rat model of cerebral IR injury. Our findings provide robust evidence that pre‐treatment with this therapeutic combination leads to marked improvements in neurological outcomes after IR insult. The observed neuroprotection appears to be closely linked to the suppression of NLRP3 inflammasome‐dependent pyroptosis and the attenuation of associated neuroinflammatory cascades. Notably, our data also implicate the upregulation of the PINK‐1/Parkin signalling axis (a central mediator of mitophagy) as a key mechanism underlying these beneficial effects. By promoting the selective clearance of dysfunctional mitochondria, enhanced mitophagy likely contributes to the preservation of neuronal integrity and resilience during the oxidative and inflammatory stress characteristic of cerebral IR injury.

Previous studies have demonstrated the neuroprotective properties of sinomenine and metformin in models of cerebral IR injury. Sinomenine exerts its effects mainly by activating the nuclear factor (erythroid‐derived 2)‐like 2 signalling pathway, enhancing antioxidant defences, reducing inflammation and suppressing NLRP3 inflammasome‐mediated pyroptosis, collectively preserving neuronal survival and blood–brain barrier integrity (Bi et al., 2021; Qiu et al., 2016). Metformin, beyond its antidiabetic action, activates AMP‐activated protein kinase signalling, promotes autophagy, reduces oxidative stress and improves mitochondrial function, thereby limiting neuronal apoptosis and infarct size (Ashabi et al., 2014; Ruan et al., 2021; Yang et al., 2016; Zhang et al., 2021; M. Zhao et al., 2019). Despite these beneficial mechanisms observed in non‐diabetic models, our study found that monotherapy with either agent did not significantly improve neurological outcomes in diabetic rats subjected to IR injury. This reduced efficacy likely reflects the multifactorial pathophysiology of diabetes, including chronic hyperglycaemia, oxidative stress, inflammation and mitochondrial dysfunction, which can impair protective signalling pathways and blunt the therapeutic effects of interventions otherwise effective in healthy or non‐diabetic animals (Mosenzon et al., 2023).

The inability of monotherapies to confer neuroprotection in our diabetic model underscores the need for more comprehensive treatment strategies. Notably, only the combined administration of sinomenine and metformin resulted in substantial neuroprotection, suggesting that the simultaneous targeting of multiple pathological pathways is necessary to overcome the detrimental effects of diabetes on the brain's response to IR injury (Lapchak & Araujo, 2007). This additive effect may arise from the complementary mechanisms of action of the two agents, with sinomenine primarily modulating inflammatory and pyroptotic pathways (Zhang et al., 2021), while metformin enhances metabolic resilience and mitochondrial quality control (Jia et al., 2015). Our findings align with the growing body of evidence suggesting that combination therapies are particularly advantageous in the context of comorbidities and complex disease states (Mokhtari, Høilund‐Carlsen et al., 2023; Mokhtari, Hosseini et al., 2023). By leveraging the additive actions of sinomenine and metformin, this dual approach holds promise for more effectively mitigating the neurological consequences of IR injury in diabetic patients.

Pyroptosis, particularly mediated by the NLRP3 inflammasome, has emerged as a central mechanism driving neuroinflammation and cellular injury following cerebral IR, with its impact being notably intensified in the context of T2DM (S. Ding et al., 2019; Gao et al., 2017). The NLRP3 inflammasome is activated through a tightly regulated sequence involving initial transcriptional upregulation of its components and subsequent assembly in response to cellular danger signals, culminating in caspase‐1 activation and pyroptotic cell death (Yu et al., 2020). A critical counter‐regulatory process to this inflammatory cascade is mitophagy, orchestrated by the PINK‐1/Parkin pathway, which safeguards neuronal health by clearing dysfunctional mitochondria and limiting the generation of reactive oxygen species. Impairment of mitophagy, as frequently observed in diabetes, further aggravates mitochondrial dysfunction and perpetuates the cycle of inflammation and cell death (Guan et al., 2018; Zhong et al., 2022).

Our study demonstrates that while sinomenine and metformin each possess mechanisms capable of modulating inflammatory and metabolic pathways, their individual administration was insufficient to overcome the profound pathophysiological disruptions present in diabetic IR injury. In contrast, the combined use of these agents produced a marked reduction in pyroptosis and oxidative stress, underscoring the necessity of a multifaceted therapeutic approach in complex disease settings. Mechanistically, our data indicate that the neuroprotective effect of sinomenine and metformin is closely tied to the restoration of PINK‐1/Parkin‐mediated mitophagy, as evidenced by the reversal of beneficial effects upon pharmacological inhibition of this pathway with Mdv‐1. Notably, Mdv‐1 alone did not significantly alter pyroptosis or inflammatory cytokines compared with the IR group, which is consistent with previous reports of its limited off‐target activity (Hong et al., 2025), thereby strengthening the conclusion that the observed effects were related to mitophagy inhibition. This highlights the pivotal role of mitochondrial quality control in modulating inflammatory cell death and suggests that therapeutic strategies aimed at both suppressing inflammasome activation and enhancing mitophagy may offer superior neuroprotection in diabetic patients at risk of cerebral IR injury.

An important aspect to consider when evaluating the novelty of our findings is how the combined use of sinomenine and metformin compares with other NLRP3 inhibitors. While selective NLRP3 inhibitors such as MCC950 have shown potent anti‐inflammatory effects, their clinical applicability remains restricted due to concerns about toxicity and off‐target actions (Das et al., 2021). In contrast, the combination of sinomenine and metformin offers broader benefits by not only attenuating NLRP3 inflammasome activation but also improving mitochondrial quality control through the regulation of mitophagy and oxidative stress. Moreover, this combination holds the potential to influence multiple parallel and distinct signalling pathways beyond NLRP3 inhibition, thereby providing a more comprehensive therapeutic strategy that warrants further investigation.

### 4.1 Limitations

This study has several limitations that should be acknowledged. First, we evaluated outcomes at a single reperfusion time point (24 h), reflecting primarily the acute phase of cerebral IR injury. While this captures peak oxidative stress, inflammasome activation, and neuronal injury, it does not provide insights into the subacute (∼7 days) or chronic (weeks to months) phases, which are clinically important for assessing long‐term neurological recovery. Future studies should include extended time points to determine whether the neuroprotective effects of combined sinomenine and metformin are sustained. Second, although we employed pharmacological inhibition of mitophagy using Mdv‐1, complementary genetic models would provide more definitive mechanistic evidence. Third, our experiments were limited to male diabetic rats, without evaluating potential sex differences or variability across different diabetic models. Additionally, as the study was conducted in rats, interspecies differences in NLRP3 inflammasome activity and mitophagy regulation may restrict direct translation to humans. Finally, while diabetes was modelled as a comorbidity, other common stroke‐associated conditions, such as hypertension or hyperlipidaemia, were not included. These factors may influence both the severity of cerebral injury and the therapeutic response. Consequently, future studies incorporating multiple comorbidities and head‐to‐head comparisons with other NLRP3‐targeting therapies are warranted to fully evaluate efficacy, optimal dosing and long‐term outcomes.

### 4.2 Conclusion

Our findings demonstrate that combined therapy with sinomenine and metformin provides significant neuroprotection against cerebral IR injury in diabetic rats. This protective effect is primarily mediated through dual modulation of NLRP3 inflammasome‐dependent pyroptosis and enhancement of PINK1/Parkin‐mediated mitophagy. In contrast to monotherapy, which showed limited efficacy, the combination treatment effectively reduced neuroinflammation and oxidative stress, highlighting the advantage of simultaneously targeting multiple pathological pathways in the diabetic brain. The abolition of these protective effects by mitophagy inhibition further underscores the critical role of mitochondrial quality control in mediating neuroprotection. These results support the potential translational value of combination therapies that address both metabolic and inflammatory dysfunctions for improving neurological outcomes in diabetic patients at risk of ischaemic brain injury.

## AUTHOR CONTRIBUTIONS

*Overall design of the project*: Wendi Li. *Conducting the experimental work and collaborating in analysing and interpreting the results*: Wendi Li, Yiying Fan and Shaik Althaf Hussain. *Assisting in drafting the manuscript*: Wendi Li, Yiying Fan and Shaik Althaf Hussain. *Supervising the whole project*: Wendi Li. Each author reviewed the final manuscript. All authors have read and approved the final version of this manuscript and agree to be accountable for all aspects of the work in ensuring that questions related to the accuracy or integrity of any part of the work are appropriately investigated and resolved. All persons designated as authors qualify for authorship, and all those who qualify for authorship are listed.

## CONFLICT OF INTEREST

The authors state that they have no conflicts of interest to declare.

## Data Availability

The datasets utilized in this study can be obtained from the corresponding author upon reasonable request.
